# Measuring walking within and outside the neighborhood in Chinese elders: reliability and validity

**DOI:** 10.1186/1471-2458-11-851

**Published:** 2011-11-09

**Authors:** Ester Cerin, Anthony Barnett, Cindy HP Sit, Man-chin Cheung, Lok-chun Janet Lee, Sai-yin Ho, Wai-man Chan

**Affiliations:** 1 Institute of Human Performance, The University of Hong Kong, 111-113 Pokfulam Rd., SAR, Hong Kong; 2 Elderly Health Service, Department of Health, Room 3502-4, 35/F Hopewell Centre, 183 Queens Road East, Wan Chai, SAR, Hong Kong; 3 School of Public Health, The University of Hong Kong, SAR, Hong Kong

## Abstract

**Background:**

Walking is a preferred, prevalent and recommended activity for aging populations and is influenced by the neighborhood built environment. To study this influence it is necessary to differentiate whether walking occurs within or outside of the neighborhood. The Neighborhood Physical Activity Questionnaire (NPAQ) collects information on setting-specific physical activity, including walking, inside and outside one's neighborhood. While the NPAQ has shown to be a reliable measure in adults, its reliability in older adults is unknown. Additionally its validity and the influence of type of neighborhood on reliability and validity have yet to be explored.

**Methods:**

The NPAQ walking component was adapted for Chinese speaking elders (NWQ-CS). Ninety-six Chinese elders, stratified by social economic status and neighborhood walkability, wore an accelerometer and completed a log of walks for 7 days. Following the collection of valid data the NWQ-CS was interviewer-administered. Fourteen to 20 days (average of 17 days) later the NWQ-CS was re-administered. Test-retest reliability and validity of the NWQ-CS were assessed.

**Results:**

Reliability and validity estimates did not differ with type of neighborhood. NWQ-CS measures of walking showed moderate to excellent reliability. Reliability was generally higher for estimates of weekly frequency than minutes of walking. Total weekly minutes of walking were moderately related to all accelerometry measures. Moderate-to-strong associations were found between the NWQ-CS and log-of-walks variables. The NWQ-CS yielded statistically significantly lower mean values of total walking, weekly minutes of walking for transportation and weekly frequency of walking for transportation outside the neighborhood than the log-of-walks.

**Conclusions:**

The NWQ-CS showed measurement invariance across types of neighborhoods. It is a valid measure of walking for recreation and frequency of walking for transport. However, it may systematically underestimate the duration of walking for transport in samples that engage in high levels of this type of walking.

## Background

There is ample empirical evidence that an active lifestyle can substantially contribute to the health and quality of life of older adults [1,2]. Due to its affordability and versatility, walking is one of the most preferred, prevalent and recommended forms of physical activity (PA) for an aging population [3,4]. In the last decade, the neighborhood built environment has been identified as an important source of influences on the walking behavior of adults as well as elders (e.g., [5-10]). However, studies exploring environment-walking relationships have failed to employ context- and/or geographically-specific measures of walking [9,11]. Walking can occur within and outside the neighborhood of residence. If we are to study the impact of the neighborhood environment on walking, it makes sense to differentiate walking that occurs within and outside the neighborhood. Additionally, since there is evidence that different environmental attributes are associated with walking for different purposes (e.g., [9,12,13], it also makes sense to differentiate utilitarian walking from walking for recreation.

The Neighborhood Physical Activity Questionnaire (NPAQ) developed by Giles-Corti and colleagues [14] aims to collect self-reported information on setting-specific PA, including walking for transportation and walking for recreation performed inside or outside one's neighborhood, defined as a 10-to-15-minute walk from home. The walking section of the NPAQ has shown adequate levels of reliability in samples of Australian [14], Canadian [15] and Belgian adults [16]. However, its reliability in older adults (65+ years) remains unknown. Since walking is the most prevalent activity in this segment of the population, it is important to examine whether the walking section of the NPAQ is appropriate for older adults.

Another issue that remains unexplored is the validity of the NPAQ. No studies have examined the correspondence of NPAQ walking estimates with objective measures of PA (e.g., pedometers or accelerometers) and activity diaries. Other self-report measures of walking were found to be substantially associated (*r *= 0.36-0.58) with step counts [4,17] and accelerometry-based weekly minutes of moderate intensity PA in older adults [18]. An analysis of the correspondence between NPAQ walking measures and activity diaries is important because objective measures of PA cannot capture information on walking purpose and geographical location (within *vs*. outside the neighborhood).

No studies have examined whether the test-retest reliability and validity of the NPAQ differ across types of neighborhoods. The NPAQ is an instrument particularly relevant to studies on environment-walking associations where context plays a major role and respondents are sampled from multiple neighborhoods with specific characteristics. Residents of walkable neighborhoods may show higher levels of reliability because they more regularly and frequently engage in walking [12,19]. Residents of areas with lower socio-economic status (SES) are likely to be less educated and have difficulties in understanding questionnaire items, which may result in a greater error of measurement when compared to their higher SES counterparts [20]. Such between-neighborhood differences in metric characteristics may yield spurious between-neighborhood differences in environment-walking associations. Thus, it is important to know whether neighborhood characteristics moderate the reliability and validity of the NPAQ.

In summary, the aims of this study were to: (1) adapt the walking section of the NPAQ for Chinese-speaking elders (hereafter named Neighborhood Walking Questionnaire - Chinese version for Seniors, NWQ-CS); (2) examine its test-retest reliability; (3) assess its validity evidence against accelerometry-based estimates of PA and daily logs of walking; and determine the moderating effects of neighborhood-level SES and transport-related walkability on reliability and validity estimates.

## Methods

This study was part of a project aimed at the development of measures for the study of environment-PA associations in Chinese elders [21]. The project was approved by the ethics committees of the Department of Health (Hong Kong SAR) and the University of Hong Kong.

### Participants and procedure

Pilot (*n *= 50) and main study (*n *= 96) samples of Chinese-speaking elders (65+ years) with no diagnosed cognitive impairment, able to walk unassisted and communicate verbally, were recruited from 32 Hong Kong neighborhoods [21,22]. The sample frame consisted of the membership lists of four Hong Kong Elderly Health Centres (EHCs) representing catchment areas of low and high transport-related walkability stratified by low and high SES (see [21] for details about the sampling procedure and Methods section below for a definition of area SES and walkability). EHCs were established by the Department of Health of the Government of Hong Kong Special Administrative Region (HKSAR) to provide membership-based comprehensive primary care services for residents aged 65 and over. EHCs members are representative of the population of Hong Kong elders in age, SES, and health status [23].

For the pilot study, gender-stratified random sampling was used to recruit approximately 12 respondents from each of the four participating EHCs (*n *= 50; response rate: 83%). A working version of the NWQ-CS was interviewer-administered after obtaining written informed consent. Participants were asked to verbalize their thoughts while answering the items. Additionally, they were asked questions about items' meaning and appropriateness of wording and format. The findings from the pilot study informed subsequent modifications of the NWQ-CS.

For the main study, participants were recruited using a two-stage sampling strategy, whereby eight street blocks with at least 25 EHC residing members were randomly selected without replacement in each of the four catchment areas. A balanced number of participants were recruited from each block via invitation letters followed up by a phone call (total *N *= 484; 15-16 participants per block; 78% response rate). After written informed consent was obtained, the NWQ-CS was interviewer-administered to the whole study sample. All participants were asked if they would consent to wearing an accelerometer for a week, keeping a log of walks, and being re-assessed on the questionnaire 2 weeks after the first assessment. From the pool of consenting participants (73% of the original 484 participants), three per street block (*n *= 96) were randomly selected to take part in this study. No significant differences were found between consenting and non-consenting participants. The socio-demographic characteristics of the samples are presented in Table 1. Low SES areas had a median monthly household income of HK$ 12,600 and 46% owner-occupiers, while high SES areas had a median income of HK$ 22,500 and 62% of owner-occupiers. The average household, intersection and commercial/service destination densities of high walkable areas were 9039, 181, and 1614 units/km^2^, respectively and those for low walkable areas were 2247, 14, and 80 units/km^2^.

**Table 1:** Characteristics of study samples

Characteristic	%	
	
	Pilot study (*n *= 50)	Main reliability/validity study (*n *= 96)
Gender		

Male	50	42

Female	50	58

Age		

65-74 years	56	62

75-84 years	36	36

85+ years	8	2

Educational attainment		

Secondary or above	42	44

Primary	46	50

No formal education but can read and write	14	7

Area of residence		

High walkable - High socio-economic status	24	26

High walkable - Low socio-economic status	24	24

Low walkable - High socio-economic status	28	24

Low walkable - Low socio-economic status	24	26

An accelerometer and a 7-day log of walks (including also a log to record accelerometer non-wear time) were delivered to the participants 8 days before the first administration of the NWQ-CS. The participants were required to wear the accelerometer for a week and at least 10 hours per day, keep a daily log of walks, and record accelerometer non-wear periods. They received a daily phone call to motivate their participation and verify compliance. On day 8 of the study, the accelerometer and log were collected. Accelerometer data were downloaded and checked to see if the daily wear-time was adequate for inclusion in subsequent analyses. Logs of walks were also checked for completeness (e.g., provision of all requested information). The NWQ-CS was interviewer-administered to those with at least five valid days of data, including a weekend day. Nine participants who failed to meet the validity criteria were asked to extend the data collection for another week and their interview was rescheduled accordingly. Ninety-four participants provided valid accelerometry (average 13.5 valid hours/day; *SD *= 1.3 hours/day) and log data. Fourteen to 20 days (average of 17 days) after the first administration of the NWQ-CS, a second assessment was conducted by the same interviewer. Ninety-two out of 96 participants participated in both assessments. Grocery vouchers were offered as incentives for participation after the successful completion of each study component (two interviews and 7-day PA monitoring).

### Measures

Data on gender, age and educational attainment were collected during the first interview. Area SES was defined using data on median household income and percentage of owner-occupiers obtained from the Census and Statistics Department of the HKSAR. Area walkability was determined using data from Centamap http://www.centamap.com and the Census and Statistics Department on household, intersection, and commercial/service destination densities expressed as units per km^2^. Area SES and walkability were dichotomized into 'high' and 'low'. Walking behavior was measured using the NWQ-CS and a 7-day log of walks. PA was objectively measured using the accelerometer MTI-ActiGraph model GT1M (Fort Walton Beach, FL, USA).

#### Neighborhood Walking Questionnaire - Chinese version for Seniors (NWQ-CS)

The NWQ-CS was based on the walking section of the NPAQ [14]. The original instrument assesses 'usual' walking within and outside the neighborhood (defined as a 10-15 min walk from home). Participants first report whether they walk within their local areas in a usual week to get to or from somewhere (i.e., for transport) or for recreation. If participants answered affirmatively, they were asked the frequency and duration (total minutes per week) of walking for transport. They were also asked to indicate the destinations to which they walked. These questions were repeated for walking for recreation, and then for walking for recreation and transport outside the neighborhood. The Chinese version of the walking section of the NPAQ (NWQ-CS) was initially translated from English to Chinese and back-translated from Chinese to English following the World Health Organization guidelines http://www.who.int/substance_abuse/research_tools/translation/en). The NWQ-CS adapted for Hong Kong seniors consists of the same items of the original instrument. However, questions about destinations are asked before those about amounts of walking. This is because the pilot study revealed that this order of questions helped the recall process. The destinations 'to/from work/study' and 'to/from school' were reworded as 'to/from work' and 'to/from school with grandchild' to make them relevant to the target population. For the purpose of this study, NWQ-CS walking data were presented as frequency and total weekly minutes of within- and outside-neighborhood walking for transport and recreation, and total weekly minutes of walking.

#### MTI-ActiGraph accelerometer model GT1M

This uniaxial accelerometer was programmed to record activity in 1-minute epochs. The participants were instructed to secure it firmly in line with the right mid-axilla, wear it during waking hours, remove it for water activities and sleep, and keep a concurrent log to record the periods of monitor wearing and non-wearing. Non-wear periods were identified using the log information as well as 100 min of consecutive zero counts, a criterion appropriate for an older population [24]. A valid day was defined as having at least 10 h of recorded activity (based on accelerometry and log data). In absence of cut-points validated in older adults, previously published cut-points originally developed for adults were used to classify activity counts into light (100-1951 counts/min), moderate (1952-5724 counts/min) and vigorous > 5724 counts/min) [25,26]. Despite their unknown validity for this age group, these cut-points were employed in earlier studies with older adults [24,27]. Data were summarized as total weekly step counts, average counts per minute, and weekly minutes of at least light (light-to-vigorous PA; LVPA) and at least moderate (moderate-to-vigorous PA; MVPA) activity.

#### Seven-day log of walks

Participants kept a 7-day log of walks undertaken outside their homes. For each walk, participants recorded the starting and finishing time and location (street), whether the location was within or outside their neighborhood of residence (defined as a 15-minute walk from home), and the purpose of the walk [errands (e.g., shopping, banking, visit to the doctor); visiting friends; go to other places (e.g., cinema, community center, park, eatery, schools); recreation; exercise; work; accompanying or picking up others]. For walks consisting of multiple destinations/stops, participants reported each walk section as a log entry. Destinations/stopping points were defined as locations where the participants spent more than 5 min. Stops at public transit points for the purpose of using public transportation were considered destinations/stops. The log was pilot-tested on a convenience sample of 10 Chinese-speaking elders balanced by gender. Walking for work was coded as work-related walking. Walking for recreation or exercise was coded as walking for recreation, while all the remaining purposes for walking were coded as walking for transport. For the purpose of this study, log data were summarized as frequency and weekly minutes of walking for transportation and recreation within and outside the neighborhood, and total weekly minutes of walking.

### Data analysis

Mean, standard deviations, medians, interquartile ranges, skewness and kurtosis were computed for all PA variables. Skewed variables were log-transformed before performing reliability and validity analyses. Test-retest reliability of each of the NWQ-CS variables was assessed using Intraclass Correlation Coefficients (ICC) based on three-level linear mixed models allowing the estimation of the proportion of total outcome variance attributable to differences between individuals (a measure of repeatability), where the total variance is defined as the sum of the within-individual (across time points), between-individual and between-area (block groups) variations. Ignoring between-area variations due to multi-stage sampling may result in an overestimation of reliability [28]. ICCs were computed for the whole sample, by area SES and by area walkability. Between-area differences in ICCs were assessed using Fisher's Z test [29]. The reliability of categorical variables was assessed using Kappa statistic and percent agreement. The latter was computed because Kappa statistics can be low and suggest poor reliability when there is little variability in the responses [30]. Standard cut-off values were used to describe the level of reliability of the examined variables, where Kappa and ICC values up to 0.40 indicated poor, > 0.40 to 0.60 moderate, > 0.60 to 0.80 substantial, and > 0.80 almost perfect [31]. Percent agreement ≥ 75% was considered adequate.

The validity of the NWQ-CS was assessed by estimating associations of NWQ-CS and corresponding accelerometry- and log-of-walks-based variables using three-level linear mixed models accounting for area clustering effects. Differences in validity estimates between areas were evaluated by including appropriate interaction terms in the regression models. Associations between NWQ-CS and other PA variables were expressed in the form of correlation coefficients, computed using the procedure outlined by Snijders and Bosker [32]. The significance of the differences in mean estimates of walking between the NWQ-CS and log of walks were examined using mixed models accounting for area level clustering effects. A probability level of 5% was adopted. Validity analyses were complemented by Bland-Altman difference plots and the estimation of associations between levels of walking, defined as the average values on the log-of-walks and NWQ-CS variables, and the difference between the NWQ-CS and log-of-walks estimates [33].

## Results

Table 2 reports the descriptive statistics for the outcome variables. Walking for transport within the neighborhood was the most prevalent activity followed by walking for recreation within the neighborhood. Walking for recreation outside the neighborhood was the least prevalent form of walking. Although the NWQ-CS and the log-of-walks yielded similar average values for walking for recreation and frequency of walking for transport, the NWQ-CS estimates of total weekly minutes of walking for transport were substantially lower than those based on the log of walks.

**Table 2:** NWQ-CS, log-of-walks, and accelerometry-based estimates of PA in a sample of Hong Kong elders

PA measure	Assessment 1		Assessment 2	
	
	Mean (SD)	Median (IQR)	Mean (SD)	Median (IQR)
*NWQ-CS (frequency/wk)*				

Walking for transport within neighborhood	10.3 (6.4)	7.0 (7.0)	10.1 (6.5)	7.0 (7.0)

Walking for transport outside neighborhood	1.8 (3.1)	0.0 (2.3)	1.8 (3.0)	0 (2.0)

Walking for recreation within neighborhood	5.6 (3.5)	7.0 (4.0)	5.7 (3.6)	7 (4.0)

Walking for recreation outside neighborhood	1.0 (2.4)	0.0 (0.0)	1.1 (2.5)	0.0 (0.0)

*NWQ-CS (min/wk)*				

Walking for transport within neighborhood	269 (254)	190 (340)	251 (247)	180 (320)

Walking for transport outside neighborhood	60 (150)	0 (75)	65 (147)	0 (70)

Walking for recreation within neighborhood	291 (251)	210 (330)	287 (243)	210 (300)

Walking for recreation outside neighborhood	57 (144)	0 (0)	65 (168)	0 (0)

Total walking	678 (404)	590 (481)	668 (401)	580 (465)

*Accelerometry-based estimates*				

Step counts (total)	60018 (24240)	58767 (35221)	-	-

MVPA (min/wk)	161 (145)	129 (187)	-	-

LVPA (min/wk)	2013 (622)	2001 (769)	-	-

Mean activity level (counts/min)	254 (107)	238 (130)	-	-

*7-day log of walks (frequency/wk)*				

Walking for transport - within neighborhood	10.3 (4.3)	9.0 (6.0)	-	-

Walking for transport - outside neighborhood	2.6 (2.3)	2.0 (3.0)	-	-

Walking for recreation - within neighborhood	5.3 (3.3)	6.0 (4.0)	-	-

Walking for recreation - outside neighborhood	1.1 (2.1)	0.0 (1.0)	-	-

*7-day log of walks (min/wk)*				

Walking for transport - within neighborhood	396 (278)	360 (285)	-	-

Walking for transport - outside neighborhood	113 (184)	0 (195)	-	-

Walking for recreation - within neighborhood	266 (203)	243 (315)	-	-

Walking for recreation - outside neighborhood	79 (169)	0 (55)	-	-

Total walking	854 (381)	813 (443)	-	-

NWQ-CS = Neighborhood Walking Questionnaire - Chinese version for Seniors; min = minutes; wk = week; PA = physical activity; MVPA = moderate-to-vigorous physical activity; LVPA = light-to-vigorous physical activity; SD = standard deviation; IQR = interquartile range

Moderate to excellent test-retest reliability was observed for the items gauging destinations from and to which respondents usually walked (Kappa ranging from 0.42 to 0.78; mean Kappa = 0.60% agreement ranging from 69% to 100%; mean percent agreement = 89%). Table 3 shows the test-retest reliability of the NWQ-CS measures of walking. As no statistically significant differences in reliability estimates between high and low SES and walkability areas were observed (all *p*s > .350), only ICCs for the whole sample are reported. In general, reliability was higher for estimates of weekly frequency than for estimates of minutes of walking, the former being substantial and the latter ranging from poor (walking for transport outside the neighborhood) to substantial (walking for recreation within neighborhood).

**Table 3:** Test-retest reliability of the NWQ-CS

Walking measure	ICC (95% CI)
*Frequency/wk*	

Walking for transport within neighborhood	0.76 (0.65, 0.82)

Walking for transport outside neighborhood	0.79 (0.54, 0.92)

Walking for recreation within neighborhood	0.78 (0.67, 0.83)

Walking for recreation outside neighborhood	0.67 (0.51, 0.80)

*Min/wk*	

Walking for transport within neighborhood	0.54 (0.39, 0.70)

Walking for transport outside neighborhood	0.37 (0.22, 0.54)

Walking for recreation within neighborhood	0.68 (0.55, 0.81)

Walking for recreation outside neighborhood	0.56 (0.40, 0.76)

Total walking	0.55 (0.41, 0.74)

NWQ-CS = Neighborhood Walking Questionnaire - Chinese version for Seniors; min = minutes; min = minutes; wk = week; ICC = intraclass correlation coefficient; 95% CI: 95% confidence intervals. Before computing the ICCs, positively skewed variables were log-transformed

Validity analyses for the NWQ-CS are summarized in Table 4. No differences in validity estimates were observed between types of neighborhood (*p*-values of area by criterion measure interaction terms > .680). Total weekly minutes of walking were moderately related to all accelerometry measures, with the weakest association being observed with MVPA. Moderate-to-strong associations were found between the NWQ-CS and log-of-walks variables, whereby there was high relative correspondence between measures of walking for recreation and moderate correspondence between measures of walking for transport. A moderate positive relationship was also found between the two instruments in relation to total weekly minutes of walking.

**Table 4:** Associations of NWQ-CS with accelerometry- and log-of-walks estimates

*NWQ-CS vs. accelerometry-based variable*	*r*	*NWQ-CS vs. accelerometry-based variable*	*r*
Total walking (min/wk) *vs*. step counts	0.48^c^	Total walking (min/wk) *vs*. LVPA (min/wk)	0.50^c^

Total walking (min/wk) *vs*. MVPA (min/wk)	0.26^a^	Total walking (min/wk) *vs*. mean activity level (counts/min)	0.53^c^

***NWQ-CS vs. log-of-walks-based variable***	***r***	***NWQ-CS vs. log-of-walks-based variable***	***r***

Walking for transport within neighborhood (frequency/wk)	0.43^c^	Walking for transport within neighborhood (min/wk)	0.56^c^

Walking for transport outside neighborhood (frequency/wk)	0.51^c^	Walking for transport outside neighborhood (min/wk)	0.41^c^

Walking for recreation within neighborhood (frequency/wk)	0.83^c^	Walking for recreation within neighborhood (min/wk)	0.68^c^

Walking for recreation outside neighborhood (frequency/wk)	0.90^c^	Walking for recreation outside neighborhood (min/wk)	0.81^c^

Total walking (min/wk)	0.44^c^		

NWQ-CS = Neighborhood Walking Questionnaire - Chinese version for Seniors; *r *= correlation coefficient; min = minutes; wk = week; PA = physical activity; MVPA = moderate-to-vigorous physical activity; LVPA = light-to-vigorous physical activity. Positively skewed variables were log-transformed. ^a ^*p *< .05; ^b ^*p *< .01; ^c ^*p *< .001

When compared with the logs of walks, the NWQ-CS yielded statistically significantly lower mean values of total walking, weekly minutes of walking for transportation, and weekly frequency of walking for transportation outside the neighborhood (Table 5). Particularly large differences were found for weekly minutes of walking for transportation. The Bland-Altman plots (not presented) revealed no obvious curvilinearity, heteroscedasticity or linear trends in the differences between the NWQ-CS estimates of walking and those of the logs of walks across levels of walking (note that all positively skewed variables had been log-transformed).

**Table 5:** Differences between NWQ-CS and log-of-walks estimates

*Variable*	Mean difference (95% CI)	
Walking for transport within neighborhood (frequency/wk)	0.1	(-1.2, 1.3)

Walking for transport outside neighborhood (frequency/wk)	-0.8^b^	(-1.3, -0.2)

Walking for recreation within neighborhood (frequency/wk)	0.3	(-0.1, 0.7)

Walking for recreation outside neighborhood (frequency/wk)	-0.1	(-0.3, 0.1)

Walking for transport within neighborhood (min/wk)	-127^c^	(-191, -63)

Walking for transport outside neighborhood (min/wk)	-53^c^	(-83, -23)

Walking for recreation within neighborhood (min/wk)	25	(-16, 65)

Walking for recreation outside neighborhood (min/wk)	-22	(-50, 7)

Total walking (min/wk)	-177^c^	(-262, -92)

NWQ-CS = Neighborhood Walking Questionnaire - Chinese version for Seniors; CI = confidence intervals; min = minutes; wk = week; ^a ^*p *< .05; ^b ^*p *< .01; ^c ^*p *< .001

## Discussion

This study examined the reliability and criterion validity of the Neighborhood Walking Questionnaire - Chinese version for Seniors (NWQ-CS), a self-report measure of walking behavior within and outside the neighborhood adapted for Chinese elders and based on the Neighborhood Physical Activity Questionnaire (NPAQ), developed by Giles-Corti and colleagues [14]. In doing so, we also examined whether area-level SES and walkability moderated the reliability and validity estimates of the NWQ-CS. No significant area moderating effects were found, providing support for the measurement invariance of the NWQ-CS across types of neighborhoods. This is in contrast to what observed in the same sample of participants with respect to the Chinese version of the International Physical Activity Questionnaire - Long, last 7-days form (IPAQ-LC), capturing domain-specific but not context-specific walking. The test-retest reliability of walking measures varied by type of neighborhood [22].

It is possible that these discrepant results were due to the NWQ-CS measuring usual patterns of walking and the IPAQ-LC focusing on walking undertaken in the last 7 days. By definition, self-reports of usual patterns of behavior should be relatively stable over short-time periods (e.g. 2-3 weeks) and minimally influenced by daily or weekly fluctuations in frequency and amounts of walking. This is less likely to be the case for 7-day recalls which, by their nature, should be affected by weekly fluctuations in behavior. When using the IPAQ-LC, residents of high walkable neighborhoods showed higher levels of test-retest reliability for transport-related walking than residents of low walkable areas [22]. This might have been due to transport-related walking patterns being more stable in the former group of residents. Walkable neighborhoods provide easy access to a variety of services and, hence, encourage regular walking for utilitarian purposes [9]. While between-neighborhood differences in regularity of walking can be theoretically captured by recall measures (e.g., IPAQ-LC, last-7-days), they are less likely to emerge when measuring customary walking behavior (e.g., NWQ-CS). This would explain why no significant differences were observed in the test-retest reliability estimates of the NWQ-CS across types of neighborhoods, but differences were observed when using the IPAQ-LC [22].

Overall, acceptable levels of test-retest reliability (ICC > 0.70; [34]) were observed for the NWQ-CS frequency measures of walking. Moderate levels of reliability were observed for measures of walking duration (min/wk). However, they were within the range of values recently reported in a sample of Canadian adults [15], despite the interval between assessments being considerably longer in the present study (average 17 days *vs*. 2.7 days). The original NPAQ reliability study found higher than the here-observed reliability estimates for all duration measures but walking for recreation outside the neighborhood [14]. The older participants' age (65-89 *vs*. 20-71 years) and longer test-retest interval periods in the present study (average 17 days *vs*. 7 days) might have been in part responsible for the differential findings.

The walking duration items of the NWQ-CS had lower test-retest reliability than those of the IPAQ long and short forms in Chinese older adults [4,22]. Elders might find it more challenging to classify various amounts of walking by context (within *vs*. outside the neighborhood) than report a total amount of walking irrespective of where it occurred due to the increased amount of cognitive processing involved. Nevertheless we maintain that, despite higher measurement errors, the NWQ-CS can still potentially provide more useful information on walking behavior than instruments that are not context specific. In fact, to identify modifiable determinants of a specific behavior, it is important to understand the context in which the behavior occurs.

To our knowledge, this is the first study to validate a version of the NPAQ. Total walking duration was significantly associated with all accelerometry-based measures. The magnitude of the association between accelerometry-based step counts and total duration of walking (*r *= 0.48) was similar to that observed in a validation study of the short form of the IPAQ in Chinese elders [4]. Interestingly, stronger associations of total walking with accelerometry-based estimates of LVPA (*r *= 0.50) than MVPA (*r *= 0.26) were observed, suggesting that the examined sample tended to walk at a slow pace, likely due to their age [18,24] but also the subtropical climate, and sometimes steep terrain of Hong Kong. A positive but slightly weaker association of walking with LVPA was observed in a recent validation study of the short form of the IPAQ in Swedish elders [18]. Unlike this study, they also found a similar association between walking and MVPA. The authors commented that their sample showed unusually high levels of activity for their age group. This observation and the differences in climate and terrain between the two geographical locations may explain the contrasting findings.

The relative correspondences (i.e., correlations) between the NWQ-CS and logs of walks were very high for both frequency and duration of walking for recreation within and outside the neighborhood (Table 4). Moreover, the differences in average estimated levels of walking for recreation between the two instruments were not statistically significantly different (Table 5). This speaks in favor of the validity of the NWQ-CS items gauging walking for recreation. The relative correspondence between the log and NWQ-CS measures of walking for transportation and total walking was moderate and only marginally acceptable [34]. Moreover, NWQ-CS estimates of walking for transport were lower than those from the log of walks (e.g., a difference of 53 min/wk or 7.6 min/day for walking outside the neighborhood). This phenomenon was not observed in the validity assessment of walking for transportation based the IPAQ-LC, last-7-days version [22].

The difference in level of validity between the NWQ-CS measure of walking for transport and walking for recreation may be due to a number of factors. Walking for recreation is usually a regular planned activity. For example, Hong Kong elders tend to regularly engage in slow-paced, early-morning walks in the neighborhood. This means that walking for recreation is likely to be less variable over short-time periods and likely to be recalled with greater accuracy. Note, for example, that in this study the test-retest reliability of walking for recreation was higher than that of walking for transport. In contrast, although walking for transport may involve some regular trips (e.g., walking with a grandchild to school), it also encompasses incidental independent or add-on journeys that can substantially vary from day to day (e.g., occasional visit to the post-office, bank, or local convenience store). The fact that a greater between-instrument difference was found between estimates of duration than frequency of walking may indicate that occasional add-on journeys may be in the main responsible for the observed differences. The NWQ-CS asks participants to report their usual levels of walking for transportation rather than recalling how much they walked for transport in the last week. These instructions may prompt participants to report only regular trips to frequently-visited destinations and leave out (at least in part) occasional trips, which may constitute a considerable portion of their total amount of walking. This would explain why in this study the NWQ-CS yielded considerably lower estimates of walking for transport than the log of walks, but not such a phenomenon was observed for the last-7-days IPAQ-LC [22].

Altogether, these findings suggest that the NWQ-CS is a valid measure of walking for recreation and frequency of walking for transport. With regards to duration of walking for transport, the NWQ-CS may systematically underestimate the actual level of walking in samples that engage in high levels of this type of walking. Nonetheless, it appears to be able to reliably pick up individual differences since the correlations between the NWQ-CS and log corresponding measures were moderate (*r *= 0.41 and 0.56) and close to the validity cut-off values considered to be acceptable (*r *= 0.50) for comparisons with diaries [34].

An additional general finding worth mentioning is the observed level of walking in the examined sample, which was much higher than that observed in recent international studies on adults (e.g., [12,13,35]) and elders (e.g., [36-38]), but similar to that previously observed in Chinese seniors [4]. These differences in levels of walking could be attributed to cultural and environmental factors. Hong Kong, as many other Chinese cities, is a very walkable city, especially in terms of utilitarian purposes. It is typified by high levels of density and land use mix, which have been shown to be related to walking for transport [19]. Moreover, less walkable areas are well connected to more walkable neighborhoods through a very efficient, affordable, and developed public transport network, which can act as a facilitator of walking for transport as well as walking for recreation outside of the neighborhood of residence [39]. Another factor that could contribute to higher levels of walking among Chinese urban dwellers is the extremely low percentage of car ownership < 35% of the total population; Census and Statistics Department, Hong Kong SAR), which is generally predictive of engagement in active transportation [40]. Finally, Chinese elders highly value an active life style and see it as an integral part of maintaining good health and social networks. Thus, if physically capable, they engage in regular physical activity, such as walking for recreation and transportation [41].

### Limitations and strengths

This study examined the reliability and validity of the NWQ-CS, an interviewer-administered measure of walking within and outside the neighborhood based on the NPAQ [14] and adapted for Chinese-speaking elders. Importantly, it is the first study to validate the walking items of a version of the NPAQ against accelerometry and log of walks. It is also the first study to assess reliability and validity differences across neighborhoods varying in SES and walkability, which is important for a proper understanding of environment-walking relationships based on data from the NPAQ.

Limitations of this study include the use of accelerometer cut-points developed for a younger population (adults) in absence of established cut-points for older adults, and the use of logs to identify walking within and outside the neighborhood. It would have been optimal to assess walking settings using Global Positioning System (GPS) monitors [42]. However, the built environment of Hong Kong with its urban canyons, steep hills, and underground walkways poses significant challenges to GPS data collection because the GPS signal is often blocked and the available satellite signals are insufficient to estimate the positioning information [43].

## Conclusions

This study provides support for the validity and reliability of the interviewer-administered NWC-CS as a measure of setting-specific walking for Chinese elders. However, caution is needed in interpreting estimates of transport-related walking duration, as the examined sample tended to underestimate the amount of walking they did. Future studies will need to explore ways to minimize the observed bias. This might include the provision of clearer instructions to respondents that highlight the need to report regular and occasional trips to destinations.

## Competing interests

The authors declare that they have no competing interests.

## Authors' contributions

EC conceptualized and coordinated the study, conceptualized the manuscript, performed all data analyses, and drafted most sections of the manuscript. AB processed physical activity data, contributed to the conceptualization and drafted some sections of the manuscript. CHPS and LCJL contributed to the translation and back-translation of the questionnaire. CHPS, WMC, SYH, MCC, LCJL, and EC contributed to the adaptation of the original English version of the questionnaire. MCC, WMC and LCJL helped co-ordinate the study and data collection. All authors made significant contributions to drafts of the manuscript and approved the final version of the manuscript.

## Pre-publication history

The pre-publication history for this paper can be accessed here:

http://www.biomedcentral.com/1471-2458/11/851/prepub
